# Mesh-Based and Meshfree Reduced Order Phase-Field Models for Brittle Fracture: One Dimensional Problems

**DOI:** 10.3390/ma12111858

**Published:** 2019-06-08

**Authors:** Ngoc-Hien Nguyen, Vinh Phu Nguyen, Jian-Ying Wu, Thi-Hong-Hieu Le, Yan Ding

**Affiliations:** 1 Institute of Research and Development, Duy Tan University, Da Nang 550000, Vietnam; 2 Department of Mathematical Sciences, School of Science, RMIT University, Melbourne, Victoria 3000, Australia; yan.ding@rmit.edu.au; 3 Department of Civil Engineering, Monash University, Clayton, Victoria 3800, Australia; 4 State Key Laboratory of Subtropical Building Science, South China University of Technology, Guangzhou 510641, China; jywu@scut.edu.cn; 5 Department of Aerospace Engineering, Ho Chi Minh City University of Technology, Ho Chi Minh City 700000, Vietnam; honghieu.le@hcmut.edu.vn

**Keywords:** phase-field theory, brittle fracture, Reduced-Order Model (ROM), Kriging model, Proper Orthogonal Decomposition (POD)

## Abstract

Modelling brittle fracture by a phase-field fracture formulation has now been widely accepted. However, the full-order phase-field fracture model implemented using finite elements results in a nonlinear coupled system for which simulations are very computationally demanding, particularly for parametrized problems when the randomness and uncertainty of material properties are considered. To tackle this issue, we present two reduced-order phase-field models for parametrized brittle fracture problems in this work. The first one is a mesh-based Proper Orthogonal Decomposition (POD) method. Both the Discrete Empirical Interpolation Method (DEIM) and the Matrix Discrete Empirical Interpolation Method ((M)DEIM) are adopted to approximate the nonlinear vectors and matrices. The second one is a meshfree Krigingmodel. For one-dimensional problems, served as *proof-of-concept demonstrations*, in which Young’s modulus and the fracture energy vary, the POD-based model can speed up the online computations eight-times, and for the Kriging model, the speed-up factor is 1100, albeit with a slightly lower accuracy. Another merit of the Kriging’s model is its non-intrusive nature, as one does not need to modify the full-order model code.

## 1. Introduction

Fracture is one of the most commonly-encountered failure modes of engineering materials and structures. Prevention of cracking-induced failure is, therefore, a major constraint in engineering designs. As with many other physical phenomena, computational modelling of fracture constitutes an indispensable tool not only to predict the failure of cracked structures, for which full-scale experiments are either too costly or even impracticable, but also to shed light onto understanding the fracture processes of many materials such as concrete, rock, ceramics, metals, biological soft tissues, etc. Within the context of continuum modelling of brittle fracture, this paper presents mesh-based and mesh-free reduced-order phase-field models. The proposed models can be used for parameter sensitivity analysis for brittle fracture problems, parameter estimation for phase-field brittle fracture models, and fracture constrained optimization problems; that is, all sorts of problems involving the repeated solution of differential equations that govern the phenomenon of brittle fracture [1].

Brittle fracture is herein modelled using a Phase-Field Model (PFM) [2,3,4], which is able to handle crack initiation, propagation, merging, and branching in two and three dimensions with a relatively simple implementation. The basic idea is to approximate a sharp crack by a diffuse band of finite width (characterized by a regularized length scale parameter *b*) via the introduction of a scalar damage-like phase-field. The crack patterns are the natural outcome of a system of two coupled partial differential equations obtained from the minimization of a potential energy that consists of a stored bulk energy, the work of external forces, and the surface energy. Furthermore, PFMs combine features of fracture mechanics (when the length scale parameter *b* approaches zero, phase-field solutions recover fracture mechanics predictions) and damage mechanics into one single theory, thus overcoming the aforementioned difficulties of many other approaches [4,5]. They have been applied to brittle fracture [3,4,6,7], quasi-brittle fracture [8,9,10,11], dynamic fracture [12,13,14,15,16], and multi-physics fracture [17,18,19,20]. We refer to the reviews of PFMs in [21,22,23] for an intensive list of references.

However, it is widely recognized that PFM simulations are very computationally demanding as fine meshes are required to resolve the highly-localized deformations within the damage band. Things can be even worse because of the “curse of dimensionality” when the system is parametrized and a higher parameter space (i.e., more varying parameters) is considered. The Reduced-Order Model (ROM) is proposed and applied in such situations. The ROM aims to reduce the dimension of the state-space system and hence to decrease computational expense, while retaining the characteristic dynamics of the original system and preserving the input-output relationship.

The general framework for model reduction, either parametric or non-parametric, is based on a projection technique. More specifically, a reduced-order model is obtained by projecting a large-scale system onto a low-dimensional subspace for which the basic vectors can be derived from the method of snapshots and Proper Orthogonal Decomposition (POD) [24,25]. For linear and non-parametric problems, the reduced-order matrices and vectors are first pre-computed, pre-projected, and remain constant, and the ROM can thus be efficiently evaluated without further reference to the original Full-Order Model (FOM) [26]. The situation becomes more complicated for nonlinear, parametric, and/or coupled systems for which the reduced-order matrices and vectors are parameter-dependent. The need for re-computing and re-projecting full-order matrices for each new parameter/newest solutions turns out to be more expensive than solving the FOM itself.

In order to address the above challenges, several approaches have been proposed, e.g., global-POD [24], POD-Greedy [27], missing point estimation [28], Gauss–Newton with approximated tensors [29], Empirical Interpolation Method (EIM) [30] and its discrete variants, the Discrete Empirical Interpolation Method (DEIM) [31,32], the Matrix Discrete Empirical Interpolation Method ((M)DEIM) [33,34,35], and matrix interpolation methods [36,37,38,39,40]. Thanks to these interpolation algorithms, especially the EIM/DEIM/(M)DEIM, model reduction for nonlinear problems has achieved significant progress in recent years.

Even though model order reduction has been applied in many fields such as fluid mechanics [24,41,42] and structural dynamics [43,44,45], there are only a few in fracture mechanics; see [46] for fatigue crack problems, [47] for softening viscoplasticity, [48,49] for the lattice model-based nonlinear fracture problems, and [50,51] for multiscale fracture problems. The aim of this paper is to present *reduced-order phase-field models for brittle fracture*, which can be used to solve parametrized brittle fracture problems efficiently, for example parameter sensitivity analysis for brittle fracture problems, parameter estimation for phase-field brittle fracture models, fracture constrained optimization problems [52], and when the randomness and uncertainty of material properties are considered. To this end, we selected the PFM of [2,3] and constructed the corresponding reduced-order models using POD-(M)DEIM and the Kriging method [53,54]. For large-scale systems, e.g., $n=10^{5}$ mesh points, the offline POD-(M)DEIM phase cannot be performed since it is impossible to store $10^{10}$-dimensional vectors. The Kriging model, as a promising alternative approach, is a meshfree surrogate model that adopts statistical methods, which can provide deeper insight into the relationship between some outputs of interest and input design variables. Compared with POD-(M)DEIM, the advantage of the Kriging model is its fast online computations and lower computer storage.

In order to avoid all the complexities of PFMs so as to focus on the ROM itself, we have selected a one-dimensional (1D) PFM for quasi-static small strain brittle fracture. In this simple setting, one does not have to deal with strain energy splits, damage boundedness, irreversibility, and so on. We emphasize that the aims of this paper are not to solve the 1D parametrized brittle fracture problem by using a ROM, the solution of which can be found analytically [55], but to demonstrate how to build ROMs for PFMs and present preliminary 1D results. The proposed ROMs are inherently multi-dimensional. The fact that we have applied them to a very simple problem of a softening bar is due to the lack of computing resources to perform the intensive offline calculations (one might need to carry out 5000 2D/3D fracture simulations). However, we are not clear if ROMs can perform well for two- and three-dimensional PFMs.

Based on computations of a one-dimensional bar with varying the Young’s modulus and fracture energy (thus, geometry, loadings, and boundary conditions are fixed even though they can also be parameters), the POD-(M)DEIM ROM can speed up the online computations eight-times, whereas for the Kriging model, the speed up factor is 1100, albeit with a slightly lower accuracy. Moreover, the Kriging model has the extra advantage of its non-intrusive nature in the sense that one does not need to modify the full-order model code. Needless to say, all these savings in the computational cost are achieved with extensive offline computations using the full model. These encouraging one-dimensional results are simply a proof-of-concept demonstration and serve as a platform to build ROMs for three-dimensional brittle fracture problems.

The remainder of this paper is structured as follows. Section 2 presents the formulation of the selected phase-field brittle fracture model, which includes the governing equations, the weak form, and the finite element solver. This is followed by Section 3, which is devoted to the presentation of the two reduced-order models: the POD-(M)DEIM ROM in Section 3.1 and the Kriging model in Section 3.2. Numerical examples are provided in Section 4 to assess the performance of these models. Conclusions and further works required to lift the limitations of the current work are given in Section 5. The POD algorithm is presented in Appendix A, and the analytical solution of the investigated model is given in Appendix B.

## 2. Phase-Field Model for Quasi-Brittle Fracture

This section briefly recalls the one-dimensional phase-field model for brittle fracture. Governing equations are given in Section 2.1, and the weak form and finite element discretization are presented in Section 2.2. We refer to [3,6,23] for details on various phase-field models for brittle and cohesive fracture.

### 2.1. Governing Equations

Let us consider a bar of length *L*, which is fixed at the left end (x=0) and pulled at the right end (x=L). Without loss of generality, a unit cross-sectional area is assumed. The displacement and crack phase-field (or damage field) are represented by the functions $u:=u(x,d;E_{0},G_{c},b,u^{*})$ and $d:=d(x,u;E_{0},G_{c},b)$, respectively. The following governing equations and boundary conditions hold [23]:

(1) $$\begin{array}{ccc} \frac{\partial \sigma }{\partial x} & =0,\; & (\mathrm{equilibrium}\;\mathrm{equation}) \end{array}$$

(2) $$\begin{array}{ccc} \left(\frac{G_{c}}{b}+E_{0}\epsilon^{2}\right)d-G_{c}b\frac{\partial^{2}d}{\partial x^{2}} & =E_{0}\epsilon^{2},\; & (\mathrm{damage}\;\mathrm{evolution}\;\mathrm{equation}) \end{array}$$

(3) $$\begin{array}{ccc} \sigma & ={\left(1-d\right)}^{2}E_{0}\epsilon ,\; & (\mathrm{stress-strain}\;\mathrm{equation}) \end{array}$$

(4) $$\begin{array}{ccc} \epsilon & =\frac{\partial u}{\partial x},\; & (\mathrm{kinematics}\;\mathrm{equation}) \end{array}$$

(5) $$\begin{array}{ccc} u(x=0) & =0,\;u\left(x=L\right)=u^{*},\; & (\mathrm{essential}\;\mathrm{boundary}\;\mathrm{condition}\;\mathrm{for}\;u) \end{array}$$

(6) $$\begin{array}{ccc} d(x=0,L) & =0,\; & (\mathrm{essential}\;\mathrm{boundary}\;\mathrm{condition}\;\mathrm{for}\;d) \end{array}$$

where ϵ(x) and σ(x) are the strain and stress fields, respectively; $E_{0}$ and $G_{c}$ denote Young’s modulus and the fracture energy of the material; *b* is the length scale introduced to approximate a sharp crack by a diffuse damage band; the loading is described via the imposed displacement $u^{*}$. The essential boundary conditions for *d* are to prevent damage from initiating at either end of the bar. For this particular phase-field model, the damage boundedness condition 0≤d≤1 is automatically satisfied [6]. As only monolithic loadings are herein considered, damage irreversibility $\dot{d}\geq 0$ is also fulfilled without any special consideration. Note however that, if needed, it can be enforced quite straightforwardly using various techniques; see [23] for details. As can be seen, PFM involves the solution of two coupled partial differential equations: the equilibrium equation and the damage evolution equation. This usually leads to a misunderstanding that PFM is just another gradient-enhanced damage model developed by [56]. It has been computationally shown in [5,11] that when the length scale approaches zero, the PFM approaches a fracture mechanics model rather than a damage model. Note that body forces are omitted for simplicity. As can be seen from the non-homogeneous Dirichlet boundary condition $u\left(x=L\right)=u^{*}$, we adopt a displacement control to trace the entire equilibrium path as snap-back does not occur for problems considered in this work. If needed, arc-length control can be used; see, e.g., [57].

### 2.2. Weak Forms and Finite Element Implementation

The weak form of the above governing equations is given by:

(7) $$\begin{array}{cc} \int_{0}^{L}\frac{\partial u}{\partial x}{\left(1-d\right)}^{2}E_{0}\frac{\partial \delta u}{\partial x}dx & =0 \end{array}$$

(8) $$\begin{array}{cc} \int_{0}^{L}\left(\frac{G_{c}}{b}+E_{0}\epsilon^{2}\right)d\delta d+G_{c}b\frac{\partial d}{\partial x}\frac{\partial \delta d}{\partial x} & =\int_{0}^{L}E_{0}\epsilon^{2}\delta ddx \end{array}$$

for test functions δu and δd.

In order to simplify the notation, let us denote the vector of state variables by x=(u,d) and the vector of model parameters by $\mu =(L,E_{0},G_{c},b,u^{*})\in \Omega$. This weak form is discretized by using standard finite elements, resulting in the following discrete equations (see Remark 1):

(9) $$\begin{array}{cc} K^{aa}\left(x;\mu \right)\;a\left(x;\mu \right) & =0, \end{array}$$

(10) $$\begin{array}{cc} K^{dd}\left(x;\mu \right)\;\bar{a}\left(x;\mu \right) & =f(x;\mu ). \end{array}$$

where $a\left(x;\mu \right)\in R^{N}$ and $\bar{a}\left(x;\mu \right)\in R^{N}$ are the nodal displacement vector and damage vector, respectively. $K^{aa}\left(x;\mu \right)\in R^{N\times N}$ and $K^{dd}\left(x;\mu \right)\in R^{N\times N}$ are the matrices, and $f\left(x;\mu \right)\in R^{N}$ is the (external) force vector (we admit this terminology is not entirely correct, but as there is only one vector in the system, we hope that it does not cause any confuse). Here, *N* is the number of grid points. Note that one has to solve the displacement equation with the constraint $a_{N}=u^{*}$ and the damage equation with ${\bar{a}}_{1}={\bar{a}}_{N}=0$. The matrices and the force vector in the above are given by:

(11) $$\begin{array}{cc} K^{aa} & =\int_{0}^{L}{\left(\nabla N\right)}^{T}E\nabla Ndx,\;E={\left(1-\bar{d}\right)}^{2}E_{0}, \end{array}$$

(12) $$\begin{array}{cc} K^{dd} & =\int_{0}^{L}\left[\left(\frac{G_{c}}{b}+E_{0}{\bar{\epsilon }}^{2}\right)N^{T}N+G_{c}b{\left(\nabla N\right)}^{T}\nabla N\right]dx, \end{array}$$

(13) $$\begin{array}{cc} f & =\int_{0}^{L}E_{0}{\bar{\epsilon }}^{2}N^{T}dx, \end{array}$$

where quantities with a bar indicate fixed values (as a staggered solver is used, which will be shortly discussed); N is the row vector of the shape functions; and ∇N denotes the row vector of the first derivatives of the shape functions. The symbol ${□}^{T}$ denotes the transpose operator.

**Remark** **1.** *Due to the non-convexity of the energy functional in terms of both displacements and damage, monolithic solvers are not very robust. That is why staggered solvers or alternating minimization solvers, where the off-diagonal coupling matrices are not needed, are popular in phase-field fracture [3,6].*

The system of Equations (9)–(10) is solved using a staggered solver, also known as the Alternating Minimization (AM) solver. That is, one fixes the damage in the equilibrium equation and solves for the displacement. Next, the updated (latest) displacement field is used to calculate the driving force and substituted into the damage evolution equation to solve for the damage field. The process is repeated until convergence. These steps are summarized in Algorithm given in Box 1, where the notation $a_{n+1}^{k}$ signifies the nodal displacement vector at load increment n+1 and AM iteration *k*; and $a_{n+1}$ denotes the converged displacement vector at step n+1. Basically, there are two computational bottlenecks:

1. the solution of two N×N systems for each AM iteration *k* and
2. the evaluation of the force vector f and matrices $K^{aa}$ and $K^{dd}$.

which render PFM computations time-consuming and may not be feasible in situations where they have to be repeatedly executed a large number of times. Reduced-Order Models (ROM) can be applied for such problems to obtain a lower order efficient model. They are introduced subsequently in Section 3. Note that we do not focus on the cost of the robust-but-slow AM solver and refer to [23] for a discussion on efficient solvers for phase-field models. We refer to Remark 2 for extension to 2D/3D problems.

Box 1 **Quasi-static brittle PFM: AM solver for load step n+1.**

1. **Initialization**: $\left(a_{n+1}^{0},{\bar{a}}_{n+1}^{0}\right)=\left(a_{n},{\bar{a}}_{n}\right)$, k=1
2. Do AM iterations: while $|{\bar{a}}_{n+1}^{k}-{\bar{a}}_{n+1}^{k-1}|>\eta \;$ ($\eta =10^{-5}$ is the precision)
  (a) **Displacement sub-problem**: solve for $a_{n+1}^{k}$ with fixed ${\bar{a}}_{n+1}^{k-1}$
    $$K^{aa}a_{n+1}^{k}=0\;\mathrm{subject}\;\mathrm{to}\;a_{n+1}^{k}\left[N\right]=u_{n+1}^{*}$$
  (b) **Phase-field sub-problem**: solve for ${\bar{a}}_{n+1}^{k}$ with fixed $a_{n+1}^{k}$
    $$\begin{array}{c} \;\;\;\;\;\;K^{dd}{\bar{a}}_{n+1}^{k}=f\;\mathrm{subject}\;\mathrm{to}\;{\bar{a}}_{n+1}^{k}\left[1\right]={\bar{a}}_{n+1}^{k}\left[N\right]=0 \end{array}$$
  (c) Set k=k+1
3. Update nodal unknowns: $\left(a_{n+1},{\bar{a}}_{n+1}\right)=\left(a_{n+1}^{k},{\bar{a}}_{n+1}^{k}\right)$

**Remark** **2.** *For hybrid isotropic brittle fracture PFMs (see, e.g., [22]), the displacement and damage sub-problems are linear. For two- (2D) or three- (3D) dimensional problems, one needs to solve an (N×nsd)×(N×nsd) system for the displacements and an N×N system for the damage where nsd is the number of spatial dimensions. Therefore, the proposed ROMs, presented in the next section, can be equally applied to 2D and 3D problems when a hybrid brittle fracture PFM is used. For tension/compression asymmetric anisotropic PFMs [6,58], the displacement sub-problem becomes nonlinear, and thus, one has to slightly modify the POD-(M)DEIM ROM as discussed in Remark 3.*

## 3. Reduced-Order Modelling

This section presents two reduced-order models, one based on the mesh-based POD method presented in Section 3.1 and the other based on a meshfree approach (cf. Section 3.2).

### 3.1. Mesh-Based Approach

Essentially, a ROM is carried out in two phases: a computationally-expensive *offline phase* and a computationally-efficient *online phase*. In the offline phase, a set of samples is collected from a standard analysis of the full-order model (in this context, the PFM simulation). This information is employed to construct a reduced-order model that is used in the online phase. In practice, the POD is applied to a set of samples collected from a full-order PFM analysis to compute a set of basis vectors (Section 3.1.1). These basis vectors are later used in the POD-DEIM to build the approximation for the force vector f (cf. Section 3.1.2) and in the POD-(M)DEIM for the matrices $K^{aa}$ and $K^{dd}$ (Section 3.1.3).

#### 3.1.1. Parameterized and Nonlinear ROM Based on the Projection Framework

Consider the system of equations in Equations (9) and (10). An ROM of this system can be obtained by approximating the full-state vectors a and $\bar{a}$ as a linear combination of *m* and $\bar{m}$ basis vectors as follows,

(14) $$a=Va_{r},\;\;\bar{a}=\bar{V}{\bar{a}}_{r},$$

where $a_{r}\in R^{m}$ and ${\bar{a}}_{r}\in R^{\bar{m}}$ are the reduced-order versions of the displacement and damage field, respectively. $V=\left[v_{1}^{u}\;v_{2}^{u}\;\ldots \;v_{m}^{u}\right]\in R^{N\times m}$ and $\bar{V}=\left[v_{1}^{d}\;v_{2}^{d}\;\ldots \;v_{\bar{m}}^{d}\right]\in R^{N\times \bar{m}}$ are the orthonormal bases. Now, at every iteration, step *k* in Box 1, projecting the system of Equations (9) and (10) onto the reduced spaces formed by these bases yields the lower order model as follows:

(15) $$\begin{array}{cc} K_{r}^{aa}\left(x^{k};\mu \right)\;a_{r} & =0, \end{array}$$

(16) $$\begin{array}{cc} K_{r}^{dd}\left(x^{k};\mu \right)\;{\bar{a}}_{r} & =f_{r}\left(x^{k};\mu \right), \end{array}$$

where the reduced-order matrices and vector are given by:

(17) $$\begin{array}{cc} K_{r}^{aa}\left(x^{k};\mu \right) & =V^{T}K^{aa}\left({\tilde{x}}^{k};\mu \right)V, \end{array}$$

(18) $$\begin{array}{cc} K_{r}^{dd}\left(x^{k};\mu \right) & ={\bar{V}}^{T}K^{dd}\left({\tilde{x}}^{k};\mu \right)\bar{V}, \end{array}$$

(19) $$\begin{array}{cc} f_{r}\left(x^{k};\mu \right) & ={\bar{V}}^{T}f\left({\tilde{x}}^{k};\mu \right). \end{array}$$

The ROM task is to find the bases V and $\bar{V}$ so that m≪N and $\bar{m}\ll N$, then to solve the system of Equations (15)–(19) using Box 1. How to determine these bases is subsequently discussed in Box 3. This task is simple and straightforward when the original system is an affine and linear system; and it would be complex for nonlinear and coupled systems. The construction and solution of the system (15)–(19) over previous solutions, i.e., $\left({\tilde{x}}^{k}=\left(Va_{r}^{k},\bar{V}{\bar{a}}_{r}^{k}\right)\right)$, and the parameter space are nontrivial. For instance, at each AM iteration *k*, the ROM requires firstly the re-construction of the full-order system matrices $K^{aa},K^{dd}$ and vector f corresponding to the parameters and previous solutions, which still depend on the dimension of the full model, and secondly, the projection of these matrices/vectors on the reduced spaces to obtain the corresponding reduced-order matrices and vector. This pure POD model may result in a longer and much more complicated computation than the original FOM. In this study, we implemented DEIM and (M)DEIM [31,35] in the offline stage to get rid of the full-dimension dependence of the ROM matrices and vector. Precisely, DEIM was used to approximate the nonlinear external force, that is the right-hand side of the damage sub-problem equation (Section 3.1.2), and (M)DEIM was adopted to approximate the nonlinear matrices (Section 3.1.3).

#### 3.1.2. DEIM

The DEIM was applied to approximate the nonlinear function of the external force in Equations (19) and (13). The idea is to select sampling of the nonlinear terms combined with interpolation among these samples to recover an approximate nonlinear evaluation, as follows:

(20) $$f_{r}\left(x^{k};\mu \right)={\bar{V}}^{T}f\left(Va_{r}^{k};\mu \right)\approx {\bar{V}}^{T}\Phi {\left(P^{T}\Phi \right)}^{-1}P^{T}f\left(Va_{r}^{k};\mu \right),$$

where matrix Φ is the POD basis vectors of the nonlinear snapshots obtained from the FOM, where $\Phi =\left[\Phi_{1},\ldots ,\Phi_{n_{f}}\right]\in R^{n\times n_{f}}$. Matrix $P=\left[e_{1},\ldots ,e_{n_{f}}\right]\in R^{n\times n_{f}}$ is the $n_{f}$-indices matrix provided by DEIM, which we used here as the original proposed in [31], where $e_{i}={\left[0,\ldots ,0,1,0,\ldots ,0\right]}^{T}\in R^{n}$ is the $i^{\mathrm{th}}$ column of the identity matrix $I_{n}\in R^{n\times n}$ for $i=1,\ldots ,n_{f}$. Note that Φ is acting as a projector of the basis on vector f (Equation (21)), while P is acting as a filter matrix that returns the non-zero components of f (Equation (22)). In other words, it is used to define the reduced mesh (see Section 3.1.3).

Let us define:

(21) $$\begin{array}{cc} D & :={\bar{V}}^{T}\Phi {\left(P^{T}\Phi \right)}^{-1}\in R^{\bar{m}\times n_{f}}, \end{array}$$

(22) $$\begin{array}{cc} F_{r}\left(a_{r}^{k};\mu \right) & :=P^{T}f\left(Va_{r}^{k};\mu \right)\in R^{n_{f}}. \end{array}$$

The POD-DEIM reduced-order of the external force in (19) now has the form:

(23) $$f_{r}\left(x^{k};\mu \right)=DF_{r}\left(a_{r}^{k};\mu \right),$$

The complexity of the evaluation of $f_{r}$ is now reduced to the evaluation of $F_{r}$ and a matrix multiplication (note that the computation of D was carried out in the offline phase). The POD-DEIM basis vectors were obtained using the algorithm in Box 2 where $N_{s}$ represents the number of sampling points (the number of points that discretize the parameter space), and $N_{T}$, in this paper, denotes the number of load increments. We refer to Box A1 for the algorithm of $\mathrm{POD}(X_{f},\epsilon_{f})$. Note that the DEIM was used in the offline phase (see Box 3) to build the reduced matrices and vectors for the approximation of the FE external force and matrices.

Box 2 **DEIM algorithm.**

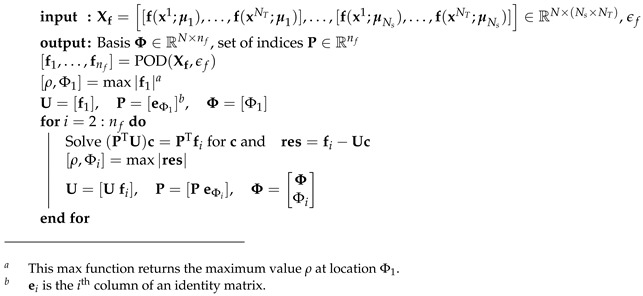

#### 3.1.3. (M)DEIM

The (M)DEIM was applied to approximate the nonlinear matrices in Equations (17) and (18). The idea is to express the nonlinear matrices (i.e., $K^{dd}\left({\tilde{x}}^{k};\mu \right)$) in vector format, then apply the DEIM to approximate it. Without loss of generality, let us consider $K^{dd}\left({\tilde{x}}^{k};\mu \right)$ (the matrix of the damage sub-problem) and define the vector version of it:

(24) $$k\left({\tilde{x}}^{k};\mu \right):=\mathrm{vec}\left(K^{dd}\left({\tilde{x}}^{k};\mu \right)\right)\in R^{N^{2}},$$

that is, k is obtained by stacking the columns of $K^{dd}$. Then, this vector is approximated as:

(25) $$k\left({\tilde{x}}^{k};\mu \right)\approx k\left(x_{r}^{k};\mu \right)=\Phi^{\bar{m}}\theta^{\bar{m}}\left(x^{k};\mu \right).$$

Here, $\Phi^{\bar{m}}\in R^{N^{2}\times m_{\bar{a}}}$ is a nonlinear-parameter-independent basis and $\theta^{\bar{m}}\left(x^{k};\mu \right)\in R^{m_{\bar{a}}}$ is the coefficient vector. Apply DEIM in Box 2 to a set of snapshots $X_{K}=\left[k\left(x^{1};\mu_{1}\right),\ldots ,k\left(x^{N_{T}};\mu_{1}\right),\ldots ,k\left(x^{N_{T}};\mu_{N_{s}}\right)\right]$ to obtain the basis $\Phi^{\bar{m}}$ and the interpolation indices $I\in R^{N^{2}}$. The reduced vector is obtained by the following projection:

(26) $$\begin{array}{cc} k_{r}\left(x_{r}^{k};\mu \right) & =\mathrm{vec}({\bar{V}}^{T}K^{dd}\left({\tilde{x}}^{k};\mu \right)\bar{V})\\ & =\left({\bar{V}}^{T}⊗{\bar{V}}^{T}\right)\Phi^{\bar{m}}\theta^{\bar{m}}\left(x^{k};\mu \right)\in R^{m_{\bar{a}}}. \end{array}$$

Let us define $D^{\bar{m}}:=\left({\bar{V}}^{T}⊗{\bar{V}}^{T}\right)\Phi^{\bar{m}}$, which is a precomputed matrix; the online computation of the reduced vector becomes:

(27) $$k_{r}\left(x_{r}^{k};\mu \right)=D^{\bar{m}}\theta^{\bar{m}}\left(x^{k};\mu \right),$$

where:

(28) $$\theta^{\bar{m}}\left(x^{k};\mu \right)={\left(\Phi_{I}^{\bar{m}}\right)}^{-1}k_{I}\left(x;\mu \right).$$

Then, the (M)DEIM approximation matrix $K^{dd}\left({\tilde{x}}^{k};\mu \right)$ is obtained by reversing the vec(·) operation. The crucial step in the online evaluation of $k_{r}$ is the computation of $k_{I}\left(x;\mu \right)$. Within the framework of the finite element method, a reduced mesh concept, also called the reduced integration domain, can be used to evaluate $k_{I}\left(x;\mu \right)$ efficiently. The basic idea is to loop over only elements belonging to I that contribute to the stiffness matrix. For more details of this technique, we refer to [35,59]. The exact same algorithm applies for $K^{aa}\left({\tilde{x}}^{k};\mu \right)$ by defining $k\left({\tilde{x}}^{k};\mu \right):=\mathrm{vec}\left(K^{aa}\left({\tilde{x}}^{k};\mu \right)\right)\in R^{N^{2}}$ and replacing $\bar{m}$ by *m* and $\bar{V}$ by V.

In summary, the offline POD-(M)DEIM is presented in Box 3. The procedure is as follows. First, $N_{s}$ sampling points in the parameter space Ω are generated, and for every point $\mu^{i}$, solve the corresponding FOM for $u^{*}\in \left[0,u_{\max}\right]$, i.e., the entire loading path. Snapshots of the nodal displacement vector, nodal damage vector, external force, and global matrices $K^{aa}$ and $K^{dd}$ for each load increment (designated by $t_{j}$) are stored. These snapshots are next used to build the bases for the POD, i.e., V and $\bar{V}$, and $\Phi^{f},\Phi^{m},\Phi^{\bar{m}}$ for POD-(M)DEIM. Finally, the DEIM (Box 2) is applied to $\Phi^{f},\Phi^{m},\Phi^{\bar{m}}$ to obtain the reduced matrices and vectors and the reduced mesh. Recall that $m_{f},m_{a},m_{\bar{a}}$ are the number of basis vectors for the force vector, matrices $K^{aa}$ and $K^{dd}$, respectively.

Once all the bases are obtained and stored, online simulations of the brittle fracture problem for any μ∈Ω can be performed using the procedure given in Box 4. We used $a_{r,n+1}^{k}$ to denote the reduced-order nodal displacement vector at load increment n+1 of AM iteration *k*. This notation also applies to the reduced nodal damage vector. As can be seen that one has to modify the FOM code to have a POD-(M)DEIM ROM code. The meshfree Kriging model, presented in the next section, is a non-intrusive technique where one does not modify the FOM code.

**Remark** **3.** *For anisotropic PFMs, the displacement sub-problem is nonlinear and most often solved with the Newton–Raphson (NR) method. For each NR iteration, one has to solve $K_{T}^{aa}\delta a=f^{ext}-f^{int}\left(a\right)$ for the displacement corrections. Therefore, in the POD-(M)DEIM, one simply applies the DEIM to the internal force vector $f^{int}\left(a\right)$ and MDEIM to the tangent matrix $K_{T}^{aa}$. This constitutes a small modification to our proposed POD algorithm. Thus, our POD ROM is inherently multi-dimensional. See [47] for an application of POD to nonlinear finite elements.*

Box 3 **Offline POD-(M)DEIM calculation.**

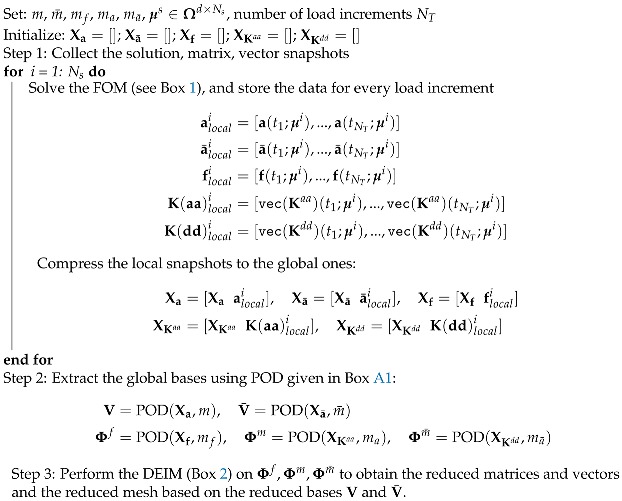

Box 4 **Phase-field ROM for quasi-static brittle fracture: online phase.**

1. **Initialization**: $\left(a_{r,n+1}^{0},{\bar{a}}_{r,n+1}^{0}\right)=\left(a_{r,n},{\bar{a}}_{r,n}\right)$, k=1
2. Do AM iterations
  (a) **Displacement sub-problem**: solve for $a_{r,n+1}^{k}$ with fixed ${\bar{a}}_{r,n+1}^{k-1}$
    (i) Solve Equation (28) on the reduced mesh to obtain $\theta^{m}\left({\bar{a}}_{r,n+1}^{k-1};\mu \right)$ (replace $\bar{m}$ by *m*).
    (ii) Reconstruct the reduce matrix $k_{r}^{aa}\left({\bar{a}}_{r,n+1}^{k-1};\mu \right)$ using Equation (27) (replace $\bar{m}$ by *m*).
    (iii) Obtain $K_{r}^{aa}\left({\bar{a}}_{r,n+1}^{k-1};\mu \right)$ by reversing the vec() operation: $K_{r}^{aa}=\mathrm{reverseVec}\left(k_{r}^{aa}\right)$.
    (iv) Solve $K_{r}^{aa}a_{r,n+1}^{k}=0$
  (b) **Phase-field sub-problem**: solve for ${\bar{a}}_{r,n+1}^{k}$ with fixed $a_{r,n+1}^{k}$
    (i) Solve Equation (28) on the reduced mesh to obtain $\theta^{\bar{m}}\left(a_{r,n+1}^{k};\mu \right)$.
    (ii) Reconstruct the reduce matrix $k_{r}^{dd}\left(a_{r,n+1}^{k};\mu \right)$ using Equation (27).
    (iii) Obtain $K_{r}^{dd}\left(a_{r,n+1}^{k};\mu \right)$ by $K_{r}^{dd}=\mathrm{reverseVec}\left(k_{r}^{dd}\right)$.
    (iv) Obtain $f_{r}\left(x^{k};\mu \right)$ using Equation (23).
    (v) Solve $K_{r}^{dd}{\bar{a}}_{r,n+1}^{k}=f_{r}$
  (c) **Set**k=k+1
3. Update nodal unknowns: $\left(a_{r,n+1},{\bar{a}}_{r,n+1}\right)=\left(a_{r,n+1}^{k},{\bar{a}}_{r,n+1}^{k}\right)$

### 3.2. Meshfree Kriging Method

**Figure 1:**
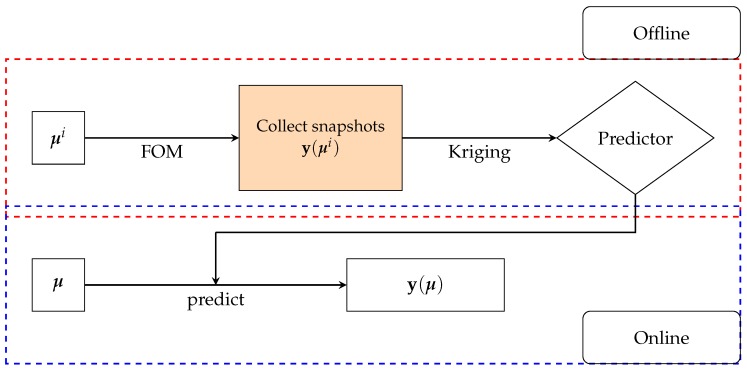
The online and offline procedures of the Kriging approach. FOM, Full-Order Model.

This section presents the Kriging model that we utilized, for which more details can be found in [53,54,60]. Generally, in order to apply the Kriging prediction model, one follows the algorithm in Figure 1. Basically, it also consists of two phases: the offline phase where data are collected by running the FOM and the predictor is built. In the online phase, the predictor is used to get the outputs for any given input. Note that this phase does not use any phase-field finite element code, resulting in a non-intrusive model (see Remark 4).

Consider an output of interest y={y(x;μ)|μ∈Ω} that varies within a parameter space Ω. Kriging models can be obtained by assuming that output y(x;μ) can be described as a linear combination of a regression model and a stochastic process as follows [60]:

(29) Y(x;μ)=m(x;μ)+Z(x;μ),

where *m* is a regression model known as the deterministic trend, which globally approximates the parameter space. The regression model is a linear combination of *p* chosen functions $f_{j}$,

(30) $$m\left(x;\mu \right)=\sum_{j=1}^{p}\beta_{j}f_{j}\left(x;\mu \right)=f{\left(x;\mu \right)}^{T}\beta ,$$

where $\beta_{j}$ are regression parameters and $f_{j}$ are regressor functions. The stochastic process *Z*, which creates the localized deviation of the parameter space, is assumed to have zero mean and variance function:

(31) $$E\left[Z\left(x;\mu_{i}\right)\times Z\left(x;\mu_{j}\right)\right]=\sigma^{2}R\left(\theta ,\mu_{i},\mu_{j}\right),$$

where $\sigma^{2}$ is variance and R() is the correlation model with parameters θ.

We now consider a set of $N_{s}$ design points denoted by $\mu_{d}=\left\{\mu_{1},\ldots ,\mu_{N_{s}}\right\}$ to which the corresponding output are $y=\{y\left(x;\mu_{1}\right),\ldots ,y\left(x;\mu_{N_{s}}\right)\}$. The regressor F with $F_{ij}=f_{j}\left(x;\mu_{i}\right)$ is given by a vector of *p* regressor functions:

(32) $$F={\left[f\left(x;\mu_{1}\right)\ldots f\left(x;\mu_{N_{s}}\right)\right]}^{T}.$$

By defining the correlation matrix R(θ) as the matrix of the stochastic process correlation between the $i^{\mathrm{th}}$ and $j^{\mathrm{th}}$ design points, we have:

(33) $$R_{ij}\left(\theta \right)=R\left(\theta ,\mu_{i},\mu_{j}\right),\;i,j=1,\ldots ,N_{s}.$$

The vector of correlations between an untried point, μ, and the design points is defined as:

(34) $$r\left(x;\mu \right)={\left[R\left(\theta ,\mu_{1},\mu \right),\ldots ,R\left(\theta ,\mu_{N_{s}},\mu \right)\right]}^{T}.$$

The predicted estimates, $\hat{y}\left(x;\mu \right)$, of the response y(x;μ) at an untried point μ, are given by:

(35) $$\hat{y}\left(x;\mu \right)=f{\left(x;\mu \right)}^{T}\hat{\beta }+r{\left(x;\mu \right)}^{T}R{\left(\theta \right)}^{-1}\left(y-F\hat{\beta }\right),$$

where $\hat{\beta }$ is estimated from the data, using least squares regression:

(36) $$\hat{\beta }={\left(F^{T}R{\left(\theta \right)}^{-1}F\right)}^{-1}F^{T}R{\left(\theta \right)}^{-1}y.$$

Once the regression model and the covariance function of a stochastic process have been determined, the prediction of y can be done by using Kriging models. According to the Design and Analysis of Computer Experiments (DACE) [60], the correlations are restricted in the form:

(37) $$R\left(\theta ,\mu_{i},\mu_{j}\right)=\prod_{k=1}^{d}R_{k}\left(\theta ,\mu_{i}^{k}-\mu_{j}^{k}\right),$$

where *d* is the dimension of the parameter space. The correlation parameters can be determined by minimizing the log-likelihood for θ as:

(38) $$\hat{\theta }=\arg\min_{\theta }[n\log\sigma^{2}+\log(|R\left(\theta \right)|)],$$

where |R(θ)| is the determinant of the correlation matrix corresponding to the design points. Assuming a Gaussian process, the optimal coefficients $\hat{\beta }$ of the correlation function solve:

(39) $$\hat{\theta }=\min_{\theta }\left|R{\left(\theta \right)}^{\frac{1}{n}}\right|\sigma^{2}.$$

The corresponding maximum likelihood estimate of the variance, $\sigma^{2}$, is:

(40) $$\sigma^{2}=\frac{1}{n}{\left(y-F\hat{\beta }\right)}^{T}\left(y-F\hat{\beta }\right).$$

The model template in the DACE toolbox (Design and Analysis of Computer Experiments) has been discussed in [61].

**Remark** **4.** *As the Kriging model is non-intrusive in the sense that one can just use a PFM code as a black box to obtain the training data, it applies to any phase-field models, namely 2D/3D and isotropic/anisotropic models.*

### 3.3. A Posteriori Error Estimations

The relative $L_{2}$-norm and Root Mean Squared Error (RMSE) used to evaluate our reduced-order models are written as:

(41) $$L_{2a}=\frac{∥a-\hat{a}{∥}_{L_{2}}}{{∥a∥}_{L_{2}}},$$

and:

(42) $$rmse_{a}=\sqrt{\frac{\sum_{i=1}^{N_{\mathrm{test}}}{\left({\hat{a}}_{i}-a_{i}\right)}^{2}}{N_{\mathrm{test}}}},$$

where $\hat{a}$ is the approximate ROM solutions and a represents the true solutions (precisely the FOM solutions). Note that $\hat{a}=Va_{r}$, so both a and $\hat{a}$ have the same length.

## 4. Numerical Examples

In this section, the performances of the two proposed ROMs are evaluated for the 1D problem stated in Section 2. Even though one can, in general, consider the variation of the geometry (*L*), material parameters ($E_{0},G_{c},b$), and loadings ($u^{*}$), we present, for simplicity, results for $\mu =\left(E_{0},G_{c}\right)\in \Omega \subset R^{2}$. That is, we consider a traction bar of a fixed length L=25, b=L/100, unit cross-section (units are deliberately left out here, given that they can be consistently chosen in any system), and that is subjected to the maximum load of $u_{\max}=37.5$. Material constants *E* and $G_{c}$ are varying parameters. Previous studies have shown that such a length scale is small enough [23] (see Remark 5). The bar is uniformly discretized with 1000 linear elements (element size h<b/2), resulting in N=1001 grid points, which produce converged results; see Appendix B for a convergence analysis. The load is applied via prescribed displacement $u^{*}\in \left[0,u_{\max}\right]$ at the right-most node. The entire loading process is divided into $N_{T}$ equal steps. The quantities of interests are (i) the displacement field, strain field, and damage field along the bar at $u_{\max}$ and (ii) the stress-strain curve, which is obtained from the load-displacement curve where load is the reaction force at the right-most node and displacement is the applied displacement $u^{*}$. These quantities computed using an FOM serve as the output of interest y=y(x;μ) used to build the Kriging predictor. All simulations were carried out using an in-house code written in MATLAB.

Concretely, we considered the parameter space $E_{0}\times G_{c}=\left[1,10\right]\times \left[1,10\right]$. The offline calculation can face the “curse of dimensionality” if we generate the samples using a full-factorial experiment. For example, let $N_{E_{0}}=N_{G_{c}}=50$ be the sample numbers of each variable; the computations require $N_{E_{0}}\times N_{G_{c}}=2500$ runs for evaluating the solution for every possible combination of every possible design value. Therefore, in this paper, we have used the Latin Hypercube Sampling method (LHS) [62] to generate statistically-optimal sampling points. The typical behaviour of this traction softening bar is as follows; see Figures 5 and 6. When the load is small, i.e., smaller than $0.8u_{\max}$, the bar is still in the elastic regime, albeit with a small damage due to the lack of an elastic domain of the chosen PFM. A further increment in loading leads to the initiation of a crack (a point in this 1D case) in the centre of the bar. Note that we have not introduced any imperfections in the bar to trigger damage localization; see, e.g., [55]. The fact that the point of damage localization is the centre of the bar is due to the perfect symmetry of the problem. The strain and damage are now localized in a small region centred at this cracking point.

**Remark** **5.** *We note that for the selected phase-field model (Section 2), the solution depends on the length scale b (see Appendix B), and one should consider its variation. However, if one adopts our length scale-insensitive PFM presented in [7], the result is independent of b.*

**Remark** **6.** *In order to make sure the ROMs can capture strain localization (or crack initiation), we have applied a large displacement of $u_{\max}=37.5$ to make sure that for all considered sampling points (i.e., all pairs of $\left(E_{0},G_{c}\right)$ used in the offline phase), damage localization happens at least once.*

We first present the ROM results in Section 4.1. That is, we generated sampling points, built various ROMs (with different accuracies), and selected the best ROM, by evaluating the ROMs with the FOM for a given random $\left(E_{0},G_{c}\right)$, to be used in online computations. The construction of the Kriging’s model was discussed in Section 4.2. Finally, Section 4.3 presents the comparison between the POD ROM and Kriging model by evaluating them against the FOM for some random values $(E_{0},G_{c})\in \Omega$.

### 4.1. ROM Results

**Figure 2:**
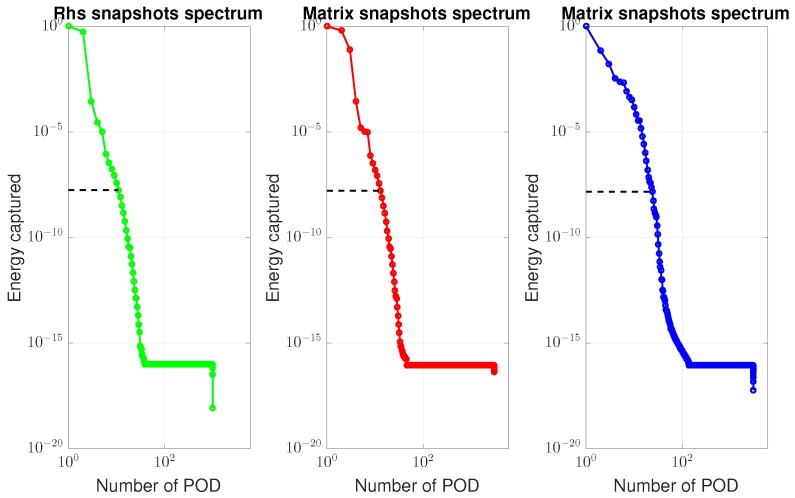
Vector and matrices’ snapshots spectrum. Left is the vector f, middle the matrix $K^{dd}$, and right the matrix $K^{aa}$.

To illustrate the performance of the POD-(M)DEIM approach, we generated $N_{s}=60$ sample points in Ω using the LHS, and built the ROM using the algorithm given in Box 3. This number of sampling points was based on our experiences with ROM; see for instance [42]. Precisely five ROMs, cf. Table 1, have been built to obtain the (M)DEIM bases of $K^{aa},K^{dd}$, and f. Figure 2 shows the snapshot spectrum and the cut-off lines corresponding to the $\epsilon_{\mathrm{POD}}=10^{-9}$ of these matrices and vector. Concretely, we have used Step 1 in the algorithm in Box A1 (Appendix A) to get the snapshot spectrum and Step 2 to get the number of required basis vectors. Table 1 presents the details of the (M)DEIM bases of each ROM. The number of M(DEIM) bases varied depending on the captured energy. More energy required a higher number of bases.

**Figure 3:**
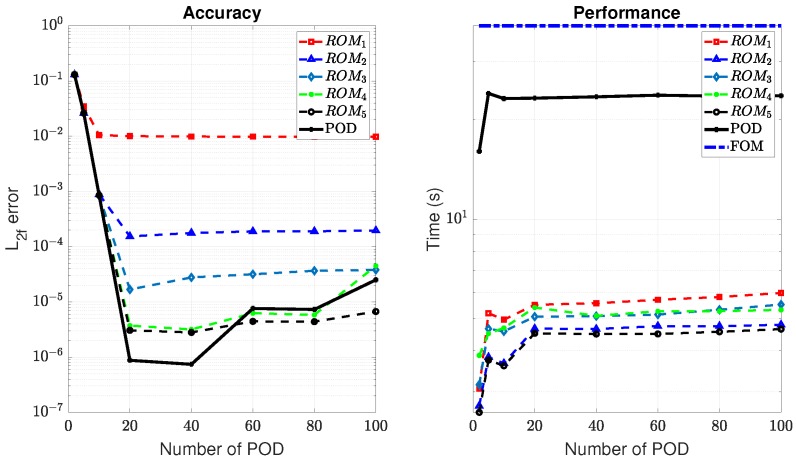
Performance of the POD-Matrix Discrete Empirical Interpolation Method ((M)DEIM) vs. pure POD. The number of POD indicates the POD of the solution vectors (*m* and $\bar{m}$).

Now, for each (M)DEIM in ROM${}_{i}$, we built the POD basis of the solution’s snapshot (displacement and damage fields) with the assumption that the number of POD bases for the displacement and and that for the damage field were equal, i.e., $m=\bar{m}$. A number of cases corresponding to m∈{2,100} were considered. The POD-(M)DEIM was then validated against the FOM and pure POD approach for a random pair of $\left(E_{0},G_{c}\right)$. Figure 3 presents the $L_{2}$-norm relative error ($L_{2f}$ for the reaction force) of ${\mathrm{ROM}}_{i}$, with i=1,…,5 and pure POD in comparison with FOM. In terms of accuracy, the pure POD performed excellently in the range of (20−40) POD basis. When more bases were used, the accuracy was reduced due to the fact that more noise and error were added into the model. Meanwhile, the accuracy of ROM${}_{i}$ increased when the captured energy increased. However, the accuracy was not much different when $\epsilon_{\mathrm{POD}}$ was in the range of $10^{-9}$ and $10^{-10}$. Furthermore, the performance of ROM${}_{i}$ was much faster than the pure POD. We note that the performance (all of the computations were performed on a PC with Intel(R) Core(TM) i7-6820HQ CPU @ 2.70 GHz 2.7 GHz, RAM 8.0 GB (64-bit operating system, x64-based processor)), in terms of both accuracy and speed, of ${\mathrm{ROM}}_{4}$ and ${\mathrm{ROM}}_{5}$ was better than ${\mathrm{ROM}}_{1},{\mathrm{ROM}}_{2}$, and ${\mathrm{ROM}}_{3}$. The reason here is that the AM solver (cf. Box 3) required iterations until convergence was satisfied. For the lower number of POD-(M)DEIM bases (meaning less accuracy of the model), it required more iterations to get convergence. For this reason, we selected ${\mathrm{ROM}}_{5}$ with m=20, $\bar{m}=20$, and $m_{a}-m_{\bar{a}}-m_{f}=28-16-14$ as the best ROM to be used in other online calculations.

### 4.2. Kriging Results

**Figure 4:**
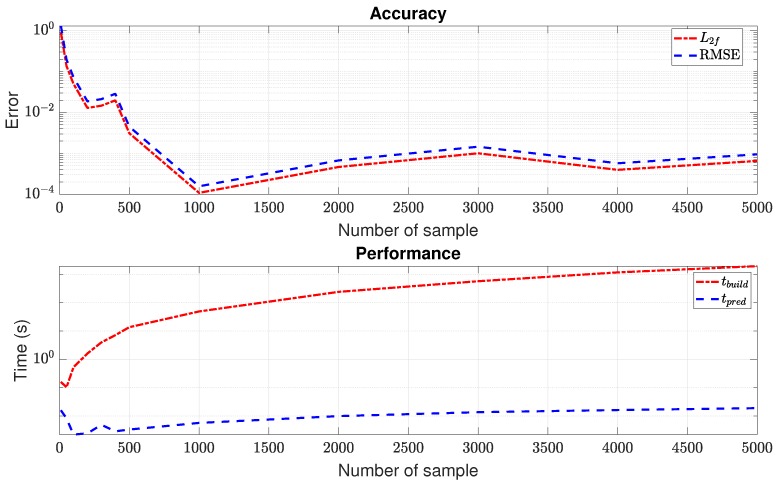
Relative errors and computational time of the Kriging model for different sampling numbers.

In order to verify the Kriging model against the sampling numbers, we generated several samples using LHS with $N_{s}$ from 10–5000, ran the FOM, and collected the output of interest, then built the Kriging model as discussed in Section 3.2; see also Figure 1. The second-order polynomial was chosen as a regression model, while the Gaussian function served as a correlation model. After that, a random pair of $\left(E_{0},G_{c}\right)$ was selected to test the Kriging’s predictor. Figure 4 shows the relative $L_{2}$-norm and RMSE of the reaction force, the construction, and the prediction time, respectively. The relative error reduced when the number of samples increased (see Remark 7 for an elaboration on this error behaviour). We note here that the $L_{2}$-norm error and the RMSE produced similar values. The prediction time was generally relatively constant; however, the model’s construction time was increased when the sampling number increased, for example, $t_{build}=0.1025$ s for $N_{s}=50$ samples; however, it increased rapidly to $t_{build}=1.17\times 10^{3}$ s for $N_{s}=4000$. From here, to avoid over-fitting, we can use $N_{s}=4000$ (instead of $N_{s}=1000$) as our Kriging model to compare with ROM. Please note here that although $t_{build}$ was considered large for $N_{s}=4000$, it was computed one time at the offline phase, so it did not affect the performance of the prediction.

**Remark** **7.** *Actually, the relative error in Figure 4 reduced significantly in the interval [1,1000] of samples, and then, it fluctuated, but did not go down to zero. From the data analysis viewpoint, the more data, the greater the accuracy of the model. However, it seemed to not be the case here. This was probably due to the discontinuity of the data (damage localization), and the Kriging model could not improve the accuracy around the discontinuity, although more data were introduced.*

### 4.3. ROM vs. Kriging

A random sample test with $N_{test}=10$ was generated to compute the relative errors of the ROM and Kriging prediction in comparison with the FOM. The averaged errors and computational time of each model are recorded and given in Table 2. Although the ROM provided more accurate solutions than Kriging (for example, relative error $L_{2f}$ of $10^{-4}$ vs. $10^{-3}$), the ROM online phase was much slower than the Kriging model. Concretely, ROM’s speedup factor, compared with FOM, was approximately eight-times, while the Kriging’s was approximately 1100.

**Figure 5:**
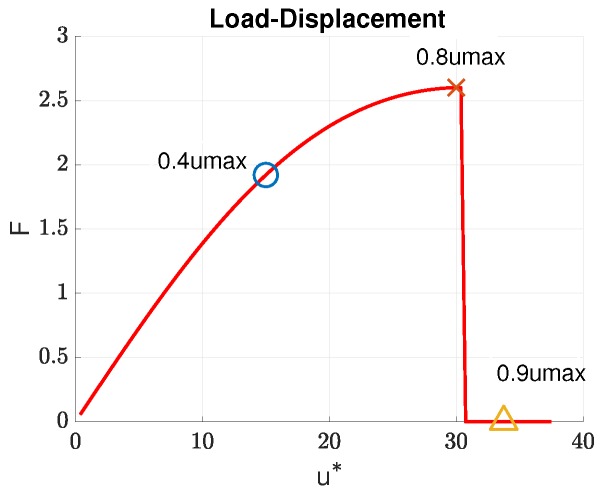
Load-displacement response of the bar for $\left(E_{0},G_{c}\right)=\left(3.705,4.332\right)$.

**Figure 6:**
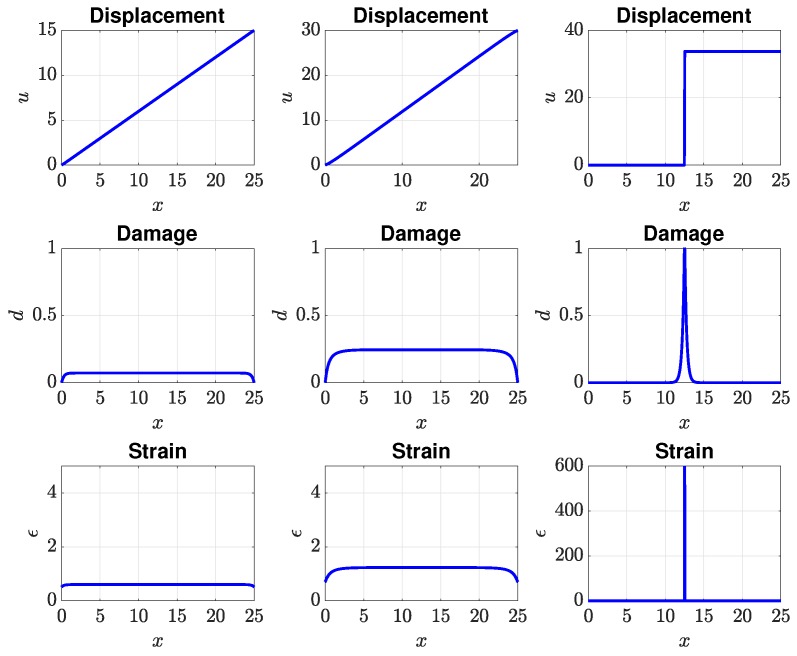
Evolution of the displacement, damage, and strain field for $\mu_{1}$: $40\%u_{\max}$ (left), $80\%u_{\max}$ (middle), $90\%u_{\max}$ (right).

We now present the mechanical behaviour of this traction bar for the case $\left(E_{0},G_{c}\right)=\left(3.705,4.332\right)$. The response of the bar, obtained using the FOM, in terms of the load-displacement curve is given in Figure 5, where *F* is the reaction force at the right-most node. As this phase-field model lacked an elastic domain, the pre-peak behaviour was not linear, as damage was non-zero immediately upon load application. When the peak was reached, the bar was suddenly broken into two parts reflected by a sharp drop in the load-displacement curve. The evolution in time of the displacement, damage, and strain field is shown in Figure 6 for three time instances: $0.4u_{\max}$, $0.8u_{\max}$, and $0.9u_{\max}$ as marked in Figure 5. Evidently, there was a strong localization of damage and strain at the middle of the bar. It is interesting to see whether ROM solutions can capture this strain localization phenomenon.

**Figure 7:**
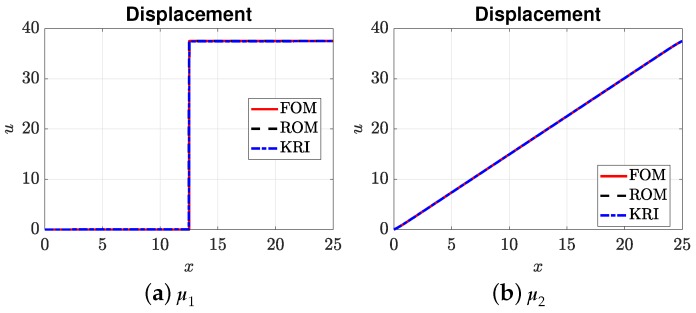
Displacement fields: for $\mu_{1}$ (corresponding to a completely broken bar) and $\mu_{2}$.

**Figure 8:**
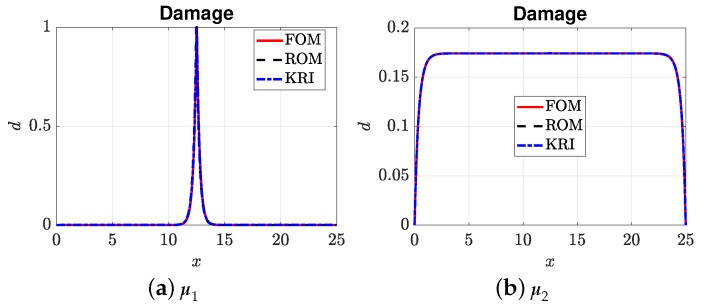
Damage fields: $\mu_{1}$ (corresponding to a completely broken bar) and $\mu_{2}$.

**Figure 9:**
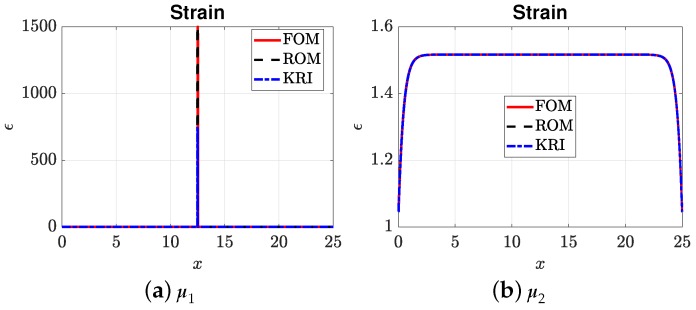
Strain field. Note that the Kriging model provided a maximum strain of only around 750 for $\mu_{1}$.

**Figure 10:**
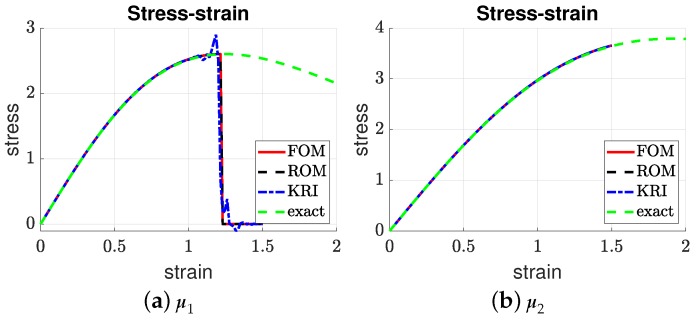
Stress-strain curve. We note the oscillations around the localization point for the Kriging model (**a**). The exact homogeneous stress-strain (valid up to crack initiation at a strain of 1.25) is given in Appendix B.

Figure 7, Figure 8, Figure 9 and Figure 10 present the output of interest: (i) the displacement field, strain field, and damage field along the bar at $u_{\max}$ and (ii) the stress-strain curve for two sets of $\mu =(E_{0},G_{c})$. Here, $\mu_{1}\;=\;\left(3.705,4.332\right)$ and $\mu_{2}=\left(3.535,9.639\right)$. In general, the mesh-based and meshfree approaches provided solutions in very good agreement with the full-order model. For $\mu_{1}$, the bar was completely broken, and thus, there was a jump in the displacement field (Figure 7a), as well as strain localizations; see Figure 8a and Figure 9a. The reason that the ROMs can capture strain localization is explained in Remark 6. For $\mu_{2}$, the damage was small, and the bar was still in the elastic regime. It can be seen that the Kriging model can capture the displacement jump (Figure 7a) and damage localization (Figure 8a), but not strain localizations, cf. Figure 9a, which involves a sudden jump of a very large magnitude.

## 5. Conclusions

Within the context of parametrized brittle fracture mechanics, we have presented two classes of reduced-order phase-field models. They can be used to carry out a very large number of computations required in different situations efficiently, ranging from parameter sensitivity analysis for brittle fracture problems, parameter estimation for phase-field brittle fracture models, to fracture constrained optimization problems. Our first ROM was a Proper Orthogonal Decomposition (POD)-based projection method that utilized the Discrete Empirical Interpolation Method (DEIM) and the Matrix Discrete Empirical Interpolation Method ((M)DEIM) to approximate the nonlinear vectors and matrices. The second was a meshfree Kriging model. For one-dimensional problems where Young’s modulus and fracture energy vary, the POD-(M)DEIM ROM can speed up the online computations eight-times, whereas the Kriging model’s speed up factor was 1100, albeit with a slightly lower accuracy. The greatest difference between the POD-(M)DEIM and Kriging model was the non-intrusive nature of the latter in the sense that one does not have to modify the full-order model code. Needless to say, all these computational savings were obtained with extensive offline computations using the full model.

Even though our reduced-order models were applied for a one-dimensional bar, we have shown that they are inherently multi-dimensional in nature. However, further works are required for 2D/3D fracture problems. It is possible that one might need to use more sampling points to capture different crack patterns. Additionally, the proposed models suffered from the following limitations

- They cannot be used for extrapolation, i.e., when the parameters are out of the bounds of the considered parameter space;
- The load has not been parametrized. That is, the maximum prescribed displacement $u_{\max}$ is fixed.
- The Kriging model resulted in oscillations around the damage localization point.

We note that the first limitation is inherent to any interpolation-based methods and might be tackled using machine learning methods. It is straightforward to overcome the second issue by just building a POD for $u_{\max}$. It is, however, very difficult to parametrize loadings in 2D and 3D. As far as the third issue is concerned, we anticipate that deep learning techniques might be helpful. For higher dimensional problems, it can become difficult. We are pursuing these paths and hope to publish them in the near future.

## Figures and Tables

**Table 1:** ROM characteristics.

$\epsilon_{\mathrm{POD}}$	$10^{-6}$ (${\mathrm{ROM}}_{1}$)	$10^{-7}$ (${\mathrm{ROM}}_{2}$)	$10^{-8}$ (${\mathrm{ROM}}_{3}$)	$10^{-9}$ (${\mathrm{ROM}}_{4}$)	$10^{-10}$ (${\mathrm{ROM}}_{5}$)
$m_{a}-m_{\bar{a}}-m_{f}$	17−6−5	22−12−10	23−12−10	27−15−13	28−16−14

**Table 2:** FOM, ROM, and KRI (Kriging) comparisons. The designation 20-20-28-16-14refers to the ROM${}_{5}$ with $m=\bar{m}=20$ and $m_{a}-m_{\bar{a}}-m_{f}=28-16-14$. Notations $L_{2f},L_{2u},L_{2d}$ represent the $L_{2}$ error for the reaction force at the right-most node, the nodal displacement, and nodal damage vector, respectively. The time in this table indicates the online computation time.

Parameter	FOM	ROM	KRI
N	1001	20-20-28-16-14	1
$t_{CPU}$ (s)	64.1	8.1	5.61 × 10${}^{-3}$
$L_{2f}$		1.74 × 10${}^{-4}$	9.63 × 10${}^{-3}$
$L_{2u}$		4.45 × 10${}^{-3}$	6.41 × 10${}^{-3}$
$L_{2d}$		2.01 × 10${}^{-3}$	8.21 × 10${}^{-3}$

**Figure A1:**
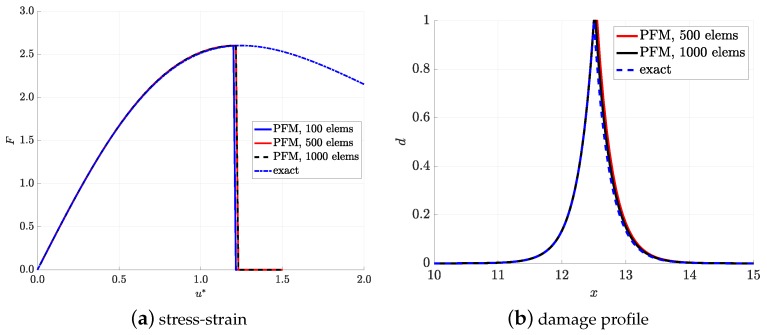
Mesh convergence analysis. PFM, Phase-Field Model.

## Appendix A. Proper Orthogonal Decomposition Algorithm

For completeness, the POD algorithm to find the basis vectors directly from the snapshot matrix $X\in R^{n\times n_{s}}$ is given in Box A1. One can input either the desired number of basic vectors (*m*) or the tolerance $\epsilon_{\mathrm{POD}}$. Note that *n* can be *N* for the displacement/damage/force snapshot or $N^{2}$ for the matrix snapshots ($K^{aa},K^{dd}$). $n_{s}$ is the number of snapshots. The total energy and capturing energy are denoted by *a* and *b*, respectively.

Box A1 POD algorithm.

## Appendix B. Exact Solutions

For the model given in Section 2, one can derive the exact solution for the homogeneous stress state, which is given by [23,55]:

(A1) $$\begin{array}{c} \sigma ={\left(\frac{G_{c}}{G_{c}+bE_{0}\epsilon^{2}}\right)}^{2}E_{0}\epsilon ,\;\sigma_{c}=\frac{3}{16}\sqrt{\frac{3E_{0}G_{c}}{b}} \end{array}$$

where $\sigma_{c}$ denotes the maximum stress, which depends on *b*. Note that the homogeneous solution is valid up to the point of damage localization or the peak load in the load-displacement curve.

We present mesh convergence analysis to justify the utilization of 1000 elements (or 1001 nodes) in all of our analyses. Three meshes consisting of 100, 500, and 1000 elements corresponding to the cases h=b, h=b/5, and h=b/10 were considered, where *h* denotes the element size. The results for the stress-strain are given in Figure A1a and the results for the damage profile in Figure A1b. As can be seen, 500 elements and 1000 elements yielded identical stress-strain data (thus, the same peak load), and 1000 elements were required to capture the cusp of the damage profile d(x)=exp(−|x|/b) [6]. Therefore, we denote the results obtained with 1000 elements’ converged solutions and using this mesh to build the ROMs. Doing so, the error is due to ROM only, and thus, it is easier to understand the ROM behaviour.
